# Outdoor Target Positioning Using Wii Remote IR Camera and Signal Modulation

**DOI:** 10.3390/s20082163

**Published:** 2020-04-11

**Authors:** Michael Lin, Kooktae Lee

**Affiliations:** Department of Mechanical Engineering, New Mexico Institute of Mining and Technology, Socorro, NM 87801, USA; michael.lin@student.nmt.edu

**Keywords:** Wii remote IR camera, outdoor target, positioning

## Abstract

This paper investigates the use of the Wii remote IR (infrared) camera for outdoor target positioning. The Wii remote IR camera is widely used in various applications because of its capability of detection of up to four IR light sources with a fast frame rate (100 Hz) and a relatively low price. However, previous applications are limited to indoor uses due to the obvious reason of sunlight interference for outdoor applications. In this paper, a signal modulation technique is introduced, which enables the IR camera to look for a particular pattern encoded in an IR beacon. In this way, the IR camera can distinguish the IR beacon from the sunlight interference. The irradiance of the sunlight reflection is also analyzed to guarantee that the IR camera can detect the IR beacon even under extremely sunny weather conditions. As the Wii remote IR camera sensor is overloaded under an extremely bright condition that blocks the camera to see any light sources, we propose the use of a filter to dim the camera. Experimental results for outdoor tests are provided to validate the proposed methods.

## 1. Introduction

**Figure 1:**
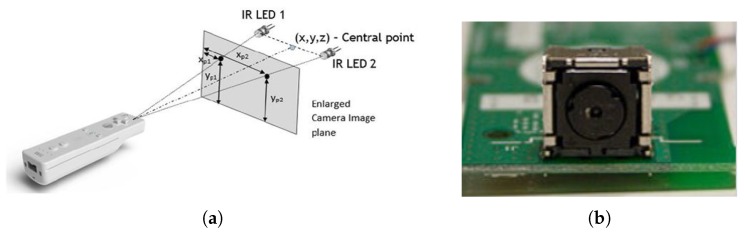
Wiimote with an Camera Image Plane (**a**) and the Camera Module (**b**).

The innovative interactive technology of the Nintendo Wii remote (or Wiimote interchangeably), shown in Figure 1a, has led to the Wii console being a game-changer in console generation with its first release in 2006. The Wiimote, a handheld device, consists of a speaker, a vibrator, an accelerometer, wireless Bluetooth connectivity, and a high-speed IR camera. The IR camera module (Figure 1b) in the Wiimote includes prebuilt-in image processing to detect up to four IR light sources with high speed (100 Hz), whereas most of the other cameras have a speed of 30 Hz, which may further down due to post-image processing. Moreover, the Wiimote camera is cost-effective compared to other high-resolution cameras.

**Figure 2:**
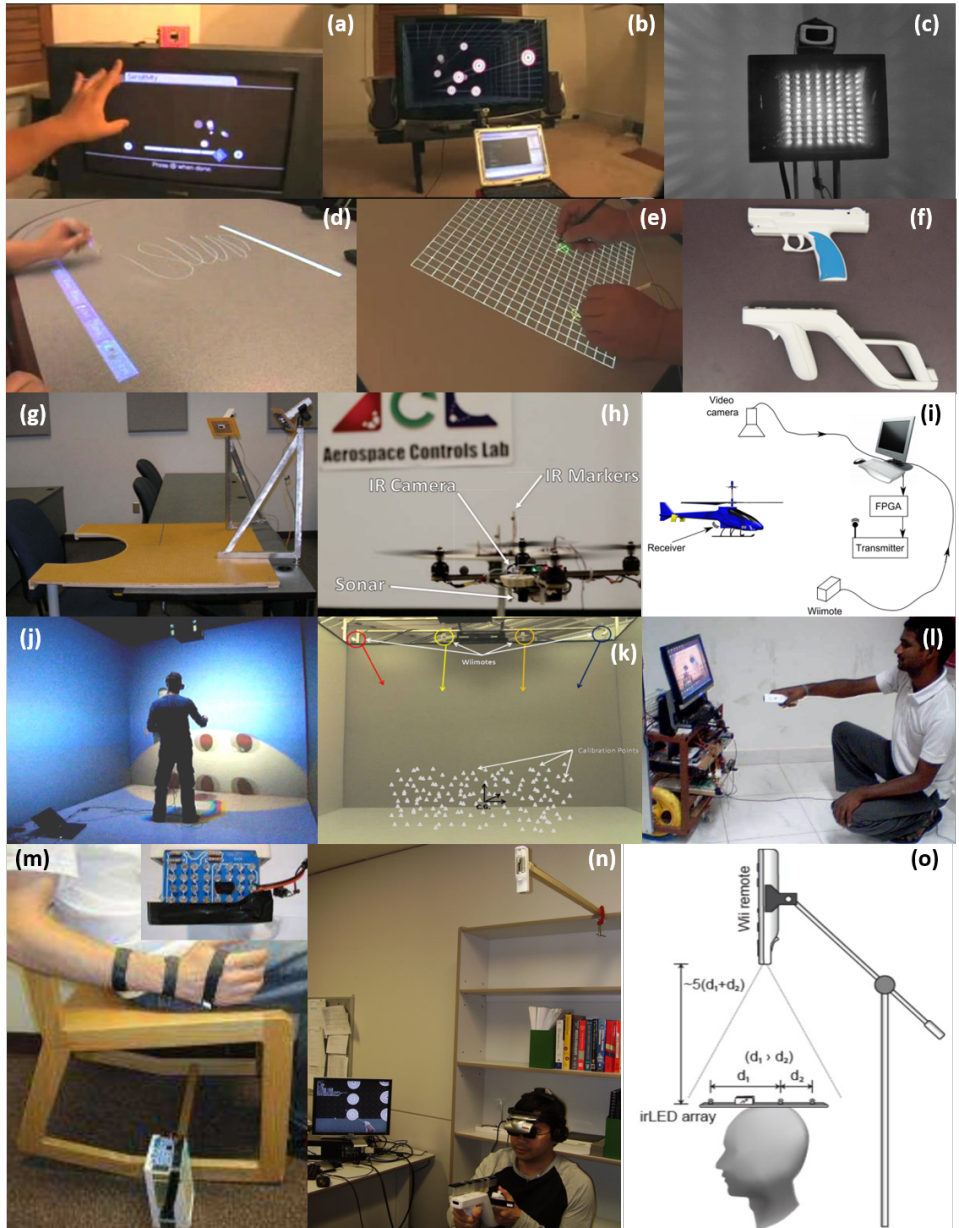
Various applications of the Wiimote: (**a**) a finger tracking; (**b**) a head tracking for VR (virtual reality) displays; (**c**) an IR-optical tracking system; (**d**) and (**e**) an interactive whiteboard; (**f**) an IR gun; (**g**) and (**m**) a rehabilitation device; (**h**) quadcopter application; (**i**) small UAV tracking; (**j**) cave-like environment VR; (**k**) automated assembly; (**l**) human-robot interaction; (**n**) head display for VR; (**o**) indoor tracking.

Aside from the Wii console’s market success, the Wii remote is widely used due to its capability for many applications including (a) finger tracking; (b) head tracking for VR (virtual reality) displays; (d), (e) an interactive whiteboard in [1]. Other applications include (c) an IR-optical tracking system [2]; (f) an IR gun [3]; (g), (m) rehabilitation devices [4,5]; (h) a quadcopter application [6]; (i) small UAV tracking [7]; (j) cave-like environment VR [8]; (k) automated assembly [9]; (l) human-robot interaction [10]; (n) head display for VR [11]; and (o) indoor head tracking [12] (see Figure 2). Despite its broad applicability, all previous Wiimote applications are quite limited to indoor uses and no existing literature is found for outdoor applications owing to the obvious reason—sunlight that contains a wide range of spectrum including IR lights. In other words, the Wiimote camera cannot differentiate between the sunlight and the target IR light source. In this paper, we aim to challenge this problem by developing a practical method that realizes the Wiimote IR camera being used for outdoor target positioning.

Previous efforts using IR light sources for object positioning include the implementation of amplitude-modulated infrared lights [13,14], where a small-size optical sensing device is developed in these studies to estimate the relative position between the sensor and active markers. Small and cheap active infrared light emitting diodes (LEDs) flickering at two different frequencies are used to measure azimuth and elevation angles inspired by visual acuity in insects. More recently, infrared-based target positioning systems are developed in [15,16,17], albeit in a slightly different way with various applications. These methods used infrared LEDs (or beacons) for accurate and robust pose estimation of a target object. Unfortunately, the aforementioned studies are all developed for indoor use and hence, it is not applicable to outdoor applications. A similar idea for outdoor target localization is introduced in [18], where ultraviolet LED markers are used instead of IR LEDs. In this research, it is claimed that the ultraviolet light in the normal sunlight is significantly less intense and thus, the natural environments are normally too dim to prevent the detection from the camera. However, no experimental results to support this argument is provided in an extreme weather condition.

To discriminate an IR beacon from sunlight interference, we introduce a signal modulation technique that generates a certain pattern encoded in the IR beacon by manipulating IR LEDs’ on/off behavior. The Wiimote IR camera only looks for this particular pattern, to distinguish it from sunlight interference. In addition to that, this paper also investigates the camera sensor overload issue, caused by an extremely bright condition. If the amount of light is more than a certain threshold, the camera’s sensor is overloaded and the camera cannot detect any IR light sources. To resolve this issue, we adopt a filter that acts as a dimmer. As no light source matches the intensity of the direct sunlight, we consider the scenario that the IR camera does not directly stare at the sun. The irradiance of the sunlight reflection is then analyzed to assure that the IR light source provides enough brightness to overcome it. The proposed signal modulation technique is developed for the Wiimote IR camera; however, it can be also applicable and extensible to any generic IR cameras that can detect a certain spectrum of IR light in wavelength. It is expected that the proposed method can be used for outdoor applications such as drone precision landing for package delivery and battery recharge in a docking station. In this case, GPS-only information is not precise enough (GPS accuracy of within 30–500 cm according to [19,20]) or some areas, such as dense urban areas with tall buildings may have poor or no GPS signals. Consequently, the proposed algorithm will be a great alternative in such a scenario.

Various experiments are conducted to validate the proposed method as well as to test its practicality. The results for different distances with two different environments are provided in the Experiments section. The result verifies that the Wiimote IR camera can be used to detect the IR light source up to 18.3 m (60 feet) away even under the sunny weather condition.

## 2. Experimental Setup

In this section, the experimental setup is introduced for target positioning using the Wiimote IR camera. This section is composed of two subsections—IR camera and IR beacon. Each subsection delivers detailed descriptions and information for each unit.

### 2.1. IR Camera

The Nintendo Wii Remote has a PixArt IR camera which has a resolution of 1024 × 768. The Wiimote includes a 128 × 96 monochrome camera with a built-in image processing that can track up to four IR light sources (or equivalently blobs). The IR camera has an I${}^{2}$C communication interface which can be easily accessed by microcontrollers. The data that can be extracted for these blobs includes the two-dimensional image plane coordinate, the size of each blob, and their intensities. The position data from the camera has the form of a byte (0–255). This needs to be converted to readable data. For locations, blobs are plotted on a two-dimensional plane with a range of 0–1023 for the *X*-axis and 0–767 for the *Y*-axis. Although the camera can track any bright object, the Wiimote has a plastic high-low pass filter in front of the camera’s lens enabling the camera to focus on detecting 940 nm of an IR light. With the filter applied and by default, the camera can detect an IR emitting object up to about 6.1 m (20 ft) away theoretically; however, this can change with the size and intensity of the light source. The camera has a viewing angle of 33 degrees horizontally and 23 degrees vertically.

#### 2.1.1. Camera Initialization

According to site WiiBrew.org [21], a wiki dedicated to homebrew on the Nintendo Wii, the Wiimote camera can be initialized by sending the data to the specified memory address through the I${}^{2}$C communication protocol. The Wiimote camera has three different modes—basic, extended, and full mode. The camera mode can be changed by sending pre-specified value to the memory address. Although each mode provides different types of data, the extended mode is used in this research, which provides *X* and *Y* locations of each of the four IR light sources as well as a rough size of them.

#### 2.1.2. Camera Sensitivity

The code for changing the camera’s default sensitivity, specifically for detecting 940 nm sources of light, requires initializing more registers. In addition to initializing the Wiimote’s IR camera, a specific value is assigned to different registers for different sensitivity settings. The formal procedure to change the sensitivity setting is provided in the Supplementary Material.

The effect of changing the camera’s sensitivity values is similar to holding a dark lens in front of the camera. The darker the lens, the less sensitive it is to other wavelengths of IR light and interference. A setting that lessens the sensitivity will improve the camera’s ability to detect 940 nm sources of light, and increase the distance the camera can see. The tradeoff is that a lower sensitivity will make the camera more prone to IR interference.

#### 2.1.3. ND (Neutral-Density) Filter

If reflections from the sun are too strong, the camera will be exposed to too much sunlight, causing the overload of image sensors. This is mainly due to the effect of overloading the IR camera’s photo-receptors. This issue cannot be resolved even with the least sensitivity setting provided above. Differently from the generic cameras as in most smartphones, the Wiimote IR camera does not include the auto-brightness feature and hence, it cannot adjust the brightness automatically. This implies that the IR camera does not detect any infrared sources if the environment is extremely bright. To avoid this issue, several tests were conducted with a grayed out piece of plastic placed in front of the IR camera. It is observed that more consistent data is collected even with extremely hot spots from sunlight reflections. With a scientific endeavor to accurately test the dimmer effect, an ND filter, a filter that reduces or modifies the intensity of all wavelengths, or colors, of light equally, is adopted. The fractional transmittance, which is the fraction of the optical power transmitted through the filter, is then computed as follows.

$$\mathrm{Fractional}\;\mathrm{transmittance}\equiv \frac{I}{I_{0}}=10^{-d},$$

where $I_{0}$ and *I* are the original and filtered intensity, respectively and *d* is an optical density determined by the ND filter ratings. For the experiments, an ND 0.3 that has the optical density d=0.3 was used. This corresponds to a 50% of the fractional transmittance when a single layer is used.

### 2.2. IR Beacon

The IR beacon consists of several clusters of IR LEDs, an electric current amplifier, and a microcontroller to control the LEDs’ blinking behavior. Not only the configuration of the IR beacon but also the required number of LEDs in the cluster is introduced based on the sunlight intensity analysis.

#### 2.2.1. IR LED and Electric Circuit

**Figure 3:**
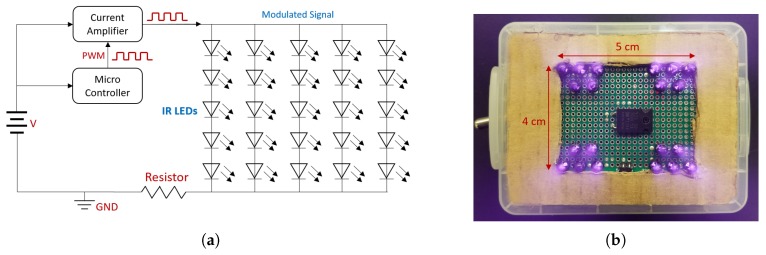
Protoype IR Beacon—total four IR blobs each of which consists of five IR LEDs. (**a**) IR Beacon Electric Circuit Diagram; (**b**) Prototype of the IR Beacon.

As the Wiimote’s IR camera tracks 940 nm light better than the other wavelengths of IR lights, the IR beacon is constructed with a large bundle of 940 nm IR LEDs. The beacon needs to be bright enough so that it can overcome the sunlight reflections’ intensity and can be detected by the IR camera. Four clusters of five LEDs are attached to a solder board as shown in Figure 3. The LEDs are positioned such that all LEDs are parallel to one another to ensure that the path of the light intersects with one another. The LEDs are controlled by the microcontroller—Arduino nano version with an electric current amplifier. Although the prototype is small enough with the size of an adult’s palm, this electric circuit can be further improved and miniaturized with electric components such as a crystal oscillator and a low-power op-amp for practicality in the future.

For reference, the specification of the LED used in this research is provided in Table 1.

#### 2.2.2. IR Beacon’s Intensity Analysis

As the Wiimote IR camera can detect up to four IR light sources, the IR beacon cannot be picked up by the IR camera, if the intensity of the IR beacon is not strong enough during outdoor experiments. Thus, it is necessary to analyze the required intensity of the IR beacon to overcome that of sunlight reflections. If the IR beacon can be seen by the Wiimote IR camera even in the worst-case scenario (i.e., when the weather is extremely sunny), then the IR camera can detect the IR beacon in any weather conditions and at any time. In what follows, the required intensity of the IR beacon is analyzed.

In everyday life, our visual reception is based on the effect produced by the light source at the surface of the surrounding objects. This means that we are interested in the amount of radiated energy, which reaches the observed surface element in a unit time. The irradiance *E* expresses radiation power Φ (flux) received by a unit area *A* of the illuminated surface with the unit of *W*/m${}^{2}$ as follows:

$$E=\frac{\mathrm{radiant}\;\mathrm{flux}\;\Phi }{\mathrm{area}\;A}.$$

**Figure 4:**
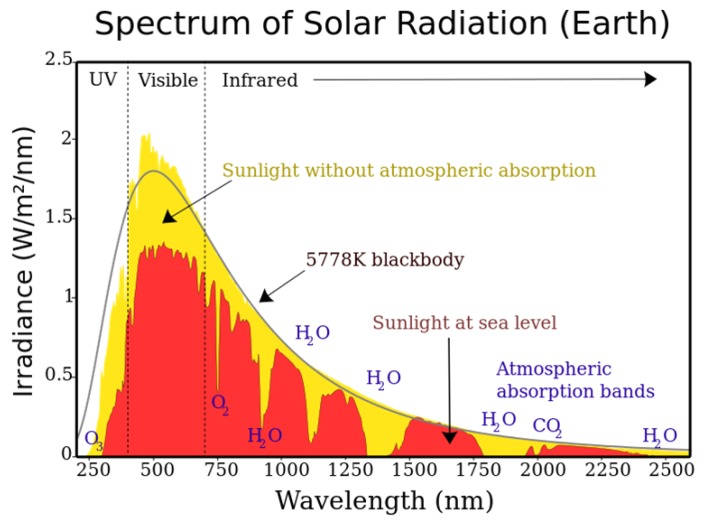
The spectrum of Solar Radiation https://en.wikipedia.org/wiki/Solar_irradiance#/media/File:Solar_spectrum_en.svg.

The spectrum of solar radiation shown in Figure 4 indicates that the sunlight irradiance for the IR wavelength of 940 nm is approximately 750 *W*/m${}^{2}$ at sea level. Some portions are absorbed by the Earth’s surface while the rest is reflected. Although various factors affect the reflectance of sunlight from the Earth’s surface, terrestrial *albedo* that describes the percentage of diffusely reflected sunlight out of the total solar radiation can be adopted to estimate the irradiance of the sunlight from the Earth’s surface. The albedo ranges between 0.1 and 0.4 in most land areas according to [22]. In the worst case that is an albedo of 0.4, the irradiance of sunlight reflection from surfaces is computed by 300W/m${}^{2}$.

From Table 1, each IR LED used here has the radiant flux of $\Phi_{\mathrm{LED}}$ = 75 m*W*, resulting in the total radiant flux of $\Phi_{\mathrm{beacon}}$ = 75 mW×20 ea =1.5W with 20 LEDs. Based on the beacon area $A_{\mathrm{beacon}}$ = 0.05 m × 0.04 m = 0.002 m${}^{2}$ from Figure 3b, the irradiance of the beacon is computed by $E=\frac{\Phi_{\mathrm{beacon}}}{A_{\mathrm{beacon}}}$ = $\frac{1.5}{0.002}$ = 750 *W*/m${}^{2}$, which is 2.5 times greater than that of the sunlight reflection from surfaces. Based on this analysis, it is guaranteed that the IR camera can at least detect the IR beacon even under the extremely bright weather condition.

## 3. Signal Modulation

The IR beacon’s intensity can be bolstered by using high-powered LEDs. However, this does not necessarily imply that the IR beacon is uniquely separated from surfaces reflecting the sunlight. A remedy proposed here is to apply a signal modulation to the IR beacon, where the signal modulation is a process of varying one or more properties of a waveform.

### 3.1. Signal Modulation

**Figure 5:**
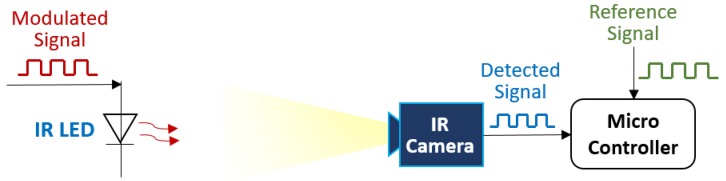
Schematic of the signal modulation for the transmitter (IR LED) and the receiver (IR camera with a Microcontroller).

By controlling the IR LEDs’ on/off behavior in the transmitter signal, a specific pattern is encoded. Then, the waveform can be read by the IR camera and decoded by a microcontroller as a serial bitstream. A schematic of the signal modulation is presented in Figure 5 to illustrate the process.

**Figure 6:**
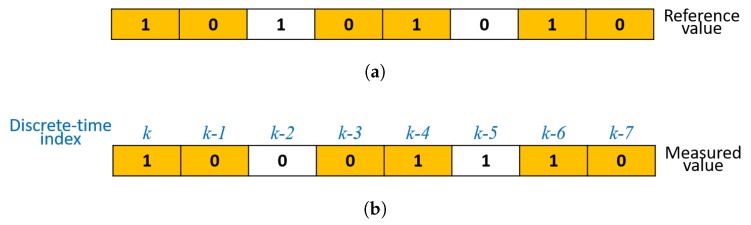
An illustration of a binary signal array for an 8-bit case. The orange color indicates the matching signal between the reference and the measured signals. (**a**) Reference binary signal array; (**b**) Measured binary signal array.

The IR camera detects modulated signals from the IR beacon—1 being HIGH and 0 being LOW for the IR beacon’s state, followed by a comparison between the measured (or detected) and the reference signal by a microcontroller. The example below shows the binary signal array that can be used to compare the reference and measured values Figure 6.

In each loop counted by a discrete-time step of the microcontroller, the previous values are shifted to the right by one and the previous right-most bit is dropped. Then, the measured signal at the current discrete-time step k∈{0,1,2,…} is saved in the left-most bit of the measured binary signal array.

Notice that the reason for shifting the previously recorded values, instead of waiting for the whole signal array to be filled with the measured value, is to maximize the comparison rate. In this case, the frequency *f* is computed by f=1/T, where *T* is the period, calculated from the loop time or the time taken between *k* and k−1. On the other hand, the frequency *f* becomes, in the case of an *n*-bit signal array, f=1/(nT) when waiting for the measured signal array being filled with *n* numbers of measurements, which will seriously slow down the frequency. Thus, the comparison can be carried out in each loop time by the proposed method.

### 3.2. Jaccard Index—A Metric to Measure Signal Matching Bits

To compare the similarity between the measured and reference array, the Jaccard index, a statistic to gauge the similarity and diversity of sample sets, is employed. The Jaccard index J(A,B) for two sets *A* and *B* is defined by $J\left(A,B\right)=\frac{\left|A\cap B\right|}{\left|A\cup B\right|}=\frac{\left|A\cap B\right|}{\left|A|+|B|-|A\cap B\right|},$ which always satisfies 0≤J(A,B)≤1. In the signal matching application, the Jaccard index can be computed as follows:

$$J\left(M,R\right)=\frac{m\;(=a\;\mathrm{total}\;\mathrm{number}\;\mathrm{of}\;\mathrm{matching}\;\mathrm{bits})}{n\;(=a\;\mathrm{total}\;\mathrm{number}\;\mathrm{of}\;\mathrm{bits}\;\mathrm{in}\;\mathrm{the}\;\mathrm{array})},$$

where *M* and *R* in J(M,R) stand for the measured and reference signal set, respectively.

One needs to set up a threshold value, $J_{\mathrm{thresh}}$, for a distinction between the IR beacon and reflections. If the current Jaccard coefficient is greater than or equal to $J_{\mathrm{thresh}}$, then the measured data can be regarded as the IR beacon. Increasing $J_{\mathrm{thresh}}$ may lead to less detectability of the beacon while decreasing $J_{\mathrm{thresh}}$ can make the IR camera more prone to interference from sunlight reflections.

### 3.3. Algorithm

A Pseudocode is provided in Algorithm 1 with the case that the reference array is given by ref = {0, 1, 0, 1, …}, i.e., alternating zero and one with each other. The most general case for an arbitrary reference array can be induced in a similar manner.
**Algorithm 1** Pseudocode for Signal Modulation   1: initialize *n*, ref[*n*], measured[*n*], m←0, *J*, $J_{\mathrm{thresh}}$   2: i←n−1
   3: **while**
i>0
**do**
   4:  measured[*i*] ← measured[i−1]
▹ Shift the data array to the right by one   5:  i←i−1   6: measured[0] ← current measured value (i.e., 0 or 1)   7: j←0   8: **while**
j<n
**do**   9:  **if** measured[*j*] == ref[*j*] **then**▹ Compare the $j^{th}$ bit 10:    m←m+1 11:  j←j+1 12: J←m/n 13: **if**
$J\geq J_{\mathrm{thresh}}$
**then**▹ Signal matching case 14:  retrieve *x* and *y* position▹ Obtain *x* and *y* position information

Several parameters are set including a total number of bits for the signal modulation array, *n*, the reference array, ref[*n*], and the measured array, measured[*n*]. Furthermore, the symbols for the Jaccard index, *J*, and the threshold value, $J_{\mathrm{thresh}}$, are assigned by the user. The integer *m* is defined for the comparison of *J* and $J_{\mathrm{thresh}}$.

The lines from 3 to 6 in Algorithm 1 is given for the update of the measured array, a size of *n*, by shifting the previous values by one and save the current measured value to measured [0]. Once again, it is unnecessary to completely fill out the measured array, which takes a total of *n* steps, leading to a waste of time and hence, a slow frequency. The proposed algorithm for the signal modulation can be used to compare the difference between the measured and reference array based on the Jaccard index at each time step with faster implementation. The bit-by-bit comparison is provided in the lines from 8 to 11 in Algorithm 1. If the Jaccard index is greater than or equal to the predefined threshold value, $J_{\mathrm{thresh}}$, then retrieve the position information because it indicates that the detected signal is the IR beacon, not the sunlight interference.

**Figure 7:**
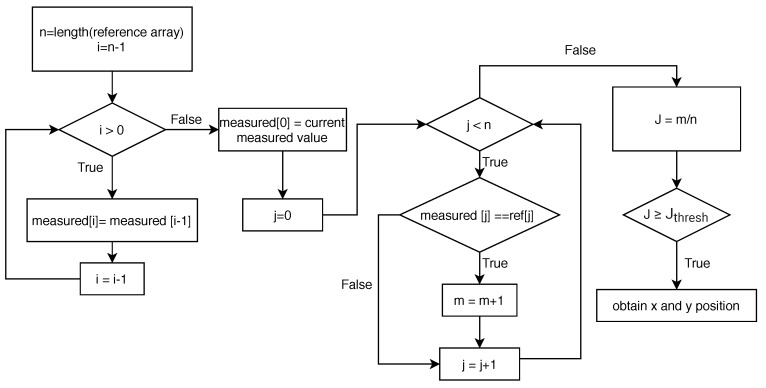
The diagram for the process of the proposed Signal Modulation algorithm.

Alternatively, a diagram showing the process of the proposed method is provided in Figure 7, to clearly describe the flow of the signal modulation algorithm.

## 4. Experiments

### 4.1. Test Environments and Conditions

**Figure 8:**
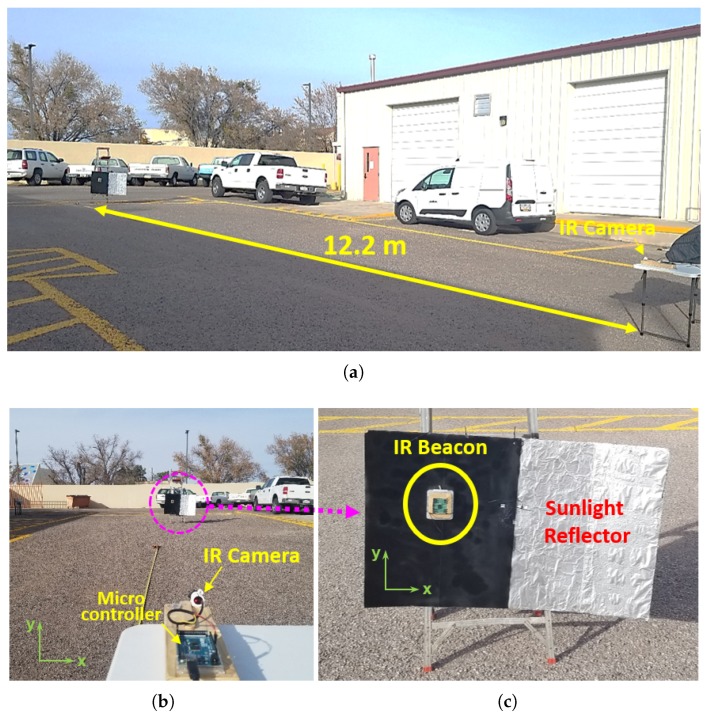
The Test Site, the IR source, and the Sunlight Reflector. (**a**) Test Site (side view); (**b**) Test Site (front view); (**c**) IR Beacon and Sunlight Reflector.

**Figure 9:**
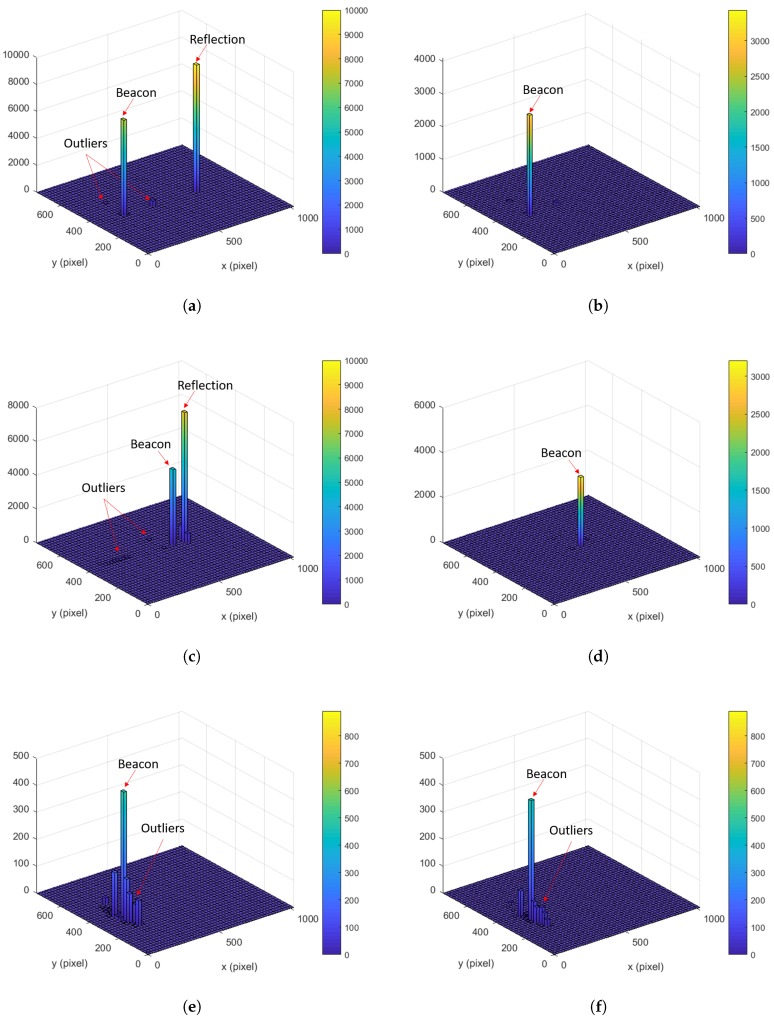
The histogram of the measured data by the IR camera for 100 s; unfiltered: (**a**), (**c**), and (**e**); filtered by the signal modulation: (**b**), (**d**), and (**f**), while varying the distance by 6.1 m (20 ft), 12.2 m (40 ft), and 18.3 m (60 ft). (**a**) 6.1 m (20 ft)/unfiltered; (**b**) 6.1 m (20 ft)/filtered; (**c**) 12.2 m (40 ft)/unfiltered; (**d**) 12.2 m (40 ft)/filtered; (**e**) 18.3 m (60 ft)/unfiltered; (**f**) 18.3 m (60 ft)/filtered.

**Figure 10:**
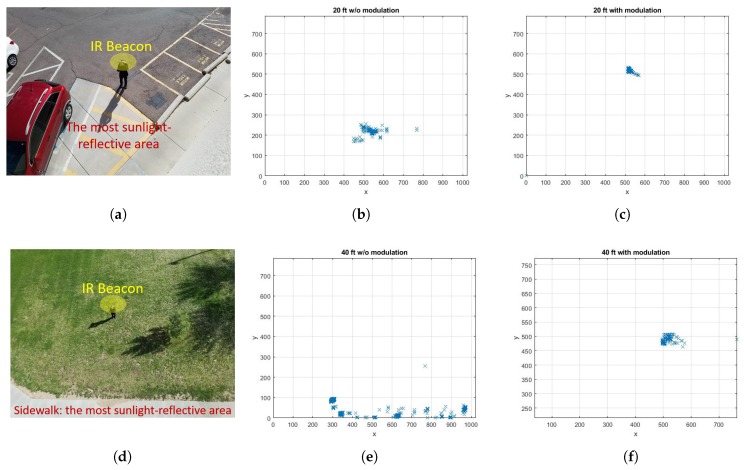
Signal modulation test result for 6.1 m (20 ft) (**a**–**c**), and 12.2 m (40 ft) (**d**–**f**). In the case of (**b**) and (**e**), the IR beacon was not placed on the test area. The test results for (**c**) and (**f**) indicate the location of the IR beacon with the signal modulation. (**a**) Testing area–6.1 m (20 ft) height; (**b**) Sunlight reflections at 6.1 m (20 ft) height; (**c**) IR beacon location at 6.1 m (20 ft) height; (**d**) Testing area–12.2 m (40 ft) height; (**e**) Sunlight reflections at 12.2 m (40 ft) height; (**f**) IR beacon location at 12.2 m (40 ft) height.

Several experiments were carried out under different environmental conditions to validate the proposed method. Two different results conducted on different days at different sites are presented from Figure 8, Figure 9 and Figure 10. The first result is for the outdoor test with various IR interference sources such as vehicles, steel walls, and asphalts as shown in Figure 8. In this scenario, the IR camera is placed parallel to the IR beacon. The second one is for the vertical test with the variation of the height by climbing up the stairs of the building.

Although the ultraviolet Index (UVI), the international standard measurement of the strength of ultraviolet radiation at a particular place and time, does not directly represent the IR intensity, it can be used to indicate how sunny it was when the experiments were carried out. According to [23], the UVI on the second experiment date was 10 on a scale of 11+. More detailed data and results with analysis are provided in the sequel.

### 4.2. Test Results

The first test is carried out in the environment surrounded by vehicles, white-painted steel walls, and asphalts as shown in Figure 8. The IR camera and the IR beacon are positioned in parallel and the distance between them is changed by 6.1 m (20 ft), 12.2 m (40 ft), and 18.3 m (60 ft). A strong sunlight reflector, made of a foil, is added right next to the IR beacon. To clearly distinguish them, the background of the IR beacon is given as a black material. In this way, one can check whether the detected signal comes from the IR beacon or the sunlight reflector.

A total of 10,000 data were collected for 100 s with three different distances. The statistic for the test is provided in Table 2. The column, #matching, implies the number of matching signal in the signal modulation. The reference array is set as ref = {0,1,0,1,0,1,0,1} with an array size 8. The Jaccard threshold value is given as $J_{\mathrm{thresh}}=0.75$, meaning the detected signal is regarded as the IR beacon if more than or equal to 6 bits in the measured array match the reference array. The column, freq., is simply #matching divided by the elapsed time. The last column, #film layers, represents the number of an ND 0.3 filter layers used in the test. In the case of 18.3 m (60 ft), eight film layers were used instead of 12 as nothing was detected by the IR camera in this case.

The histogram for the measured data is presented in Figure 9. The left and right figure, respectively, corresponds to the same distance analysis for the unfiltered case (without the signal modulation) and the filtered case (with the signal modulation). Although there was a strong interference from the sunlight reflector, it is observed that the IR camera can detect the IR beacon (two peaks: one from the IR beacon and another from the sunlight reflector). This is because the irradiance of the IR beacon is also strong enough to be detected by the IR camera according to the given analysis in Section 2.2.2. The reason that the value for the IR beacon is smaller than that for the reflection is due to the blinking of the IR beacon.

As shown in Figure 9, almost all outliers and interference are effectively filtered out by the signal modulation. The proposed signal modulation technique with the irradiance analysis is thus proven to be practical for the outdoor target positioning.

The second test was conducted to validate the proposed method with the IR camera facing down and the IR beacon placed at the ground level. The testing occurred around the times of 11 AM to 4 PM for the extreme condition, with no clouds blocking or refracting the sun. Figure 10 shows the human operator at the ground level surrounded by (a) asphalt and a vehicle while the IR camera was located at 6.1 m (20 ft) height and (d) grasses and a sidewalk with the IR camera at 12.2 m (40 ft). The asphalt, vehicles, and sidewalk provided a large number of reflections as indicated in Figure 10b,e, where the IR beacon was not located. With the implementation of the signal modulation, the IR camera was successfully able to eliminate the sunlight interference and localize and track the IR beacon as shown in Figure 10c,f.

## 5. Conclusions

In this paper, we investigated the use of the Wiimote IR camera for outdoor target positioning. To avoid interference from sunlight reflections, the signal modulation technique is proposed. This enables the IR camera to filter out the interference, leading to the successful detection of the IR beacon. The irradiance of the sunlight reflection was also analyzed for the IR beacon to overcome it. Furthermore, an ND filter was introduced for the camera sensor overload issue. To validate the proposed method, several test results are provided. From the experimental results, it is proven that the proposed method can be used to detect an outdoor target up to 18.3 m (60 ft) away from the IR camera even in sunny weather conditions. The frequency of the target detection is achieved more than 30 Hz for both 6.1 m (20 ft) and 12.2 m (40 ft) cases. Thus, it is shown that the proposed methods are practical in terms of low-cost, fast, and computationally efficient outdoor target positioning. This leaves open the possibility of the proposed signal modulation algorithm being implementable to broad applications, where accurate positioning of an outdoor target is necessarily required such as drone precision landing.

## Figures and Tables

**Table 1:** The specification of the IR LED (SFH 4544 Infrared Emitter 940 nm by OSRAM Opto Semiconductors) used in experiments.

No	Parameter	Symbol	Values	Unit
1	Centroid wavelength	$\lambda_{\mathrm{centroid}}$	940	nm
2	Half angle	φ	±10	deg
3	Forward voltage	$V_{F}$	1.6	V
4	Forward current	$I_{F}$	100	mA
5	Total radiant flux	Φ	75	mW

**Table 2:** Outdoor data statistics with the variation of the distance of the IR beacon.

No	Distance	Measurement Counts	Elapsed Time	#Matching	Freq.	#Film Layers
1	6.1 m (20 ft)	10,000	100 s	3433	34.33 Hz	12
2	12.2 m (40 ft)	10,000	100 s	3206	32.06 Hz	12
3	18.3 m (60 ft)	10,000	100 s	889	8.89 Hz	8

## Supplementary Materials

The following are available online at https://www.mdpi.com/1424-8220/20/8/2163/s1, Description S1: Wii remote IR camera sensitivity setup.
