# Supplementary material for: Outdoor Target Positioning Using Wii Remote IR Camera and Signal Modulation
Source: Sensors (Basel). 2020 Apr 11;20(8):2163. doi: 10.3390/s20082163 (PMC7218719; doi:10.3390/s20082163)
Supplement: Supplementary file 1 [file sensors-20-02163-s001.pdf]

sensors

MDPI

Article

Outdoor Target Positioning using Wii Remote IR Camera and Signal Modulation

Michael Lin † and **Kooktae Lee** *,†

Department of Mechanical Engineering, New Mexico Institute of Mining and Technology; michael.lin@student.nmt.edu
* Correspondence: kooktae.lee@nmt.edu; Tel.: +1-575-835-5554
† Current address: New Mexico Institute of Mining and Technology, Socorro, NM 87801, USA.

Received: 06 February 2020; Accepted: 07 April 2020; Published: date

check for updates

Abstract: This paper investigates the use of the Wii remote IR (infrared) camera for outdoor target positioning. The Wii remote IR camera is widely used in various applications because of its capability of detection of up to four IR light sources with a fast frame rate (100 Hz) and a relatively low price. However, previous applications are limited to indoor uses due to the obvious reason of sunlight interference for outdoor applications. In this paper, a signal modulation technique is introduced, which enables the IR camera to look for a particular pattern encoded in an IR beacon. In this way, the IR camera can distinguish the IR beacon from the sunlight interference. The irradiance of the sunlight reflection is also analyzed to guarantee that the IR camera can detect the IR beacon even under extremely sunny weather conditions. As the Wii remote IR camera sensor is overloaded under an extremely bright condition that blocks the camera to see any light sources, we propose the use of a filter to dim the camera. Experimental results for outdoor tests are provided to validate the proposed methods.

Keywords: Wii remote IR camera; outdoor target; positioning

Camera Sensitivity Setup

The Wiimote camera has three different modes – basic, extended, and full mode. The camera mode can be changed by sending pre-specified value to the memory address. Although each mode provides different types of data, the extended mode is used in this research, which provides X and Y locations of each of the four IR light sources as well as a rough size of them.

The code for changing the camera's default sensitivity, specifically for detecting 940nm sources of light, requires initializing more registers. In addition to initializing the Wiimote's IR camera, a specific value (variables p0, p1, p2, p3) is assigned to different registers for different sensitivity settings. The formal procedure to change the sensitivity is provided below.

1) Write data 0x01 to control register address 0x30.
2) Write data 0x02, 0x00, 0x00, 0x71, 0x01, 0x00, p0 to control register address 0x00 (write 7 bytes).
3) Write control register Write data 0x00, p1 to address 0x07 (write 2 bytes).
4) Write data p2, p3 to control register address 0x1A (write 2 bytes).
5) For control register address 0x33 Write data 0x03.
6) Write 0x08 to control register address 0x30 When registering multiple bytes, the register address is automatically incremented, so it can be written continuously.

Below are values for sensitivity variables (p0, p1, p2, p3) according to Wiibrew[1].

[1] https://wiibrew.org/wiki/Wiimote

1) Sensitivity Setting 1: p0 = 0x72, p1 = 0x20, p2 = 0x1F, p3 = 0x03
2) Sensitivity Setting 2: p0 = 0xC8, p1 = 0x36, p2 = 0x35, p3 = 0x03
3) Sensitivity Setting 3: p0 = 0xAA, p1 = 0x64, p2 = 0x63, p3 = 0x03
4) Sensitivity Setting 4: p0 = 0x96, p1 = 0xB4, p2 = 0xB3, p3 = 0x04
5) Sensitivity Setting 5: p0 = 0x96, p1 = 0xFE, p2 = 0xFE, p3 = 0x05

where the prefix '0x' is used to denote that it is a hexadecimal number.

The effect of changing the camera's sensitivity values is similar to holding a dark lens in front of the camera. The darker the lens, the less sensitive it is to other wavelengths of IR light and interference. A setting that lessens the sensitivity will improve the camera's ability to detect 940nm sources of light, and increase the distance the camera can see. The tradeoff is that a lower sensitivity will make the camera more prone to IR interference.

Although five different sensitivity settings are available, no document was found to explain how these values were chosen. From numerous outdoor tests it turns out that the best sensitivity values in this study are empirically obtained by p0 = 0x96, p1 = 0xfd, p2 = 0x40, p3 = 0x00.

© 2020 by the authors. Licensee MDPI, Basel, Switzerland. This article is an open access article distributed under the terms and conditions of the Creative Commons Attribution (CC BY) license (http://creativecommons.org/licenses/by/4.0/).
